# Robotic and Plastic Surgery: actuality and prospects for the near future, a scoping review

**DOI:** 10.31744/einstein_journal/2024RW0710

**Published:** 2024-04-17

**Authors:** Vitor Pelogi Arienzo, Dov Charles Goldenberg, Marcos Antonio Neves Noronha, Phellipe Fabrini Santos Lucas, Beatriz Peral Venet Ferreira, Tatiana Scarparo de Oliveira

**Affiliations:** 1 Hospital Israelita Albert Einstein São Paulo SP Brazil Hospital Israelita Albert Einstein, São Paulo, SP, Brazil.; 2 Hospital das Clínicas Faculdade de Medicina Universidade de São Paulo São Paulo SP Brazil Hospital das Clínicas, Faculdade de Medicina, Universidade de São Paulo, São Paulo, SP, Brazil.

**Keywords:** Robotic surgical procedures, Surgery, plastic, Microsurgery, Minimally invasive surgical procedures

## Abstract

**Objective:**

This work aims to review the existing use of robotics in plastic surgery.

**Methods:**

A meticulous selection process identified 22 articles relevant to this scoping review.

**Results:**

The literature on the use of robotics in plastic surgery is sparse. Nonetheless, this review highlights emerging benefits in microsurgery, breast reconstruction, and transoral surgery.

**Conclusion:**

This scoping review identifies critical articles reporting the emerging use of robotics in plastic surgery. While the scientific medical community has yet to extensively document its use, the available evidence suggests a promising future for robotics in this field.

## INTRODUCTION

The recent decades have witnessed notable improvements in surgical techniques compounded by reduced incision size and recovery time. Specifically, the advent of laparoscopy in the 1980s and the introduction of surgery assisted by automated pulley systems in the early 21^st^ century have been critical in paving the way for Robotic Surgery to become a reality. More recently, the COVID-19 pandemic has highlighted the increasing need for remote access, making Robotic Surgery an ideal and valuable approach to meeting patients’ surgical needs.^(1)^

Robotic Surgery is gaining popularity, particularly within the field of Plastic Surgery. Although Robotic Surgery is still in its early days, it is being readily adopted. Advantages include reduced tremors, reduced incision size, improved ergonomics for the surgical team, the potential for remote surgeries, and the ability to move in and manipulate the surgical field in all 360 degrees with precision.^(2)^

NASA and the United States Armed Forces initially funded research in robotic surgery to facilitate remote operations in hard-to-reach environments, such as space. Although these remote surgery options have yet to become a reality in such hostile environments, their potential has been well recognized.^(2)^

The number of robot-assisted plastic surgeries reported in the literature has been limited. Published studies include breast reconstruction, facial reconstruction, raising and insetting microsurgical flaps in transoral approaches, microneural surgery for brachial plexus reconstruction, robotic lymphovenous anastomosis for lymphedema surgery, and body contouring surgery.^(3-5)^

A 2022 opinion survey among plastic surgeons found that most had positive views on robotic surgery and believed in its potential to improve surgical outcomes.^(6)^ Overall, 89.7% of respondents endorsed the integration of robotics into the future of plastic surgery, particularly in pelvic/perineal reconstruction (56.4%), abdominal reconstruction (46.5%), microsurgery (43.6%), and supermicrosurgery (44.2%). The same study also reviewed existing literature to identify the advantages and disadvantages of this approach compared to conventional surgery.^(6)^ Despite the positive feedback for Robotic Surgery, this study also identified cost and complexity as critical barriers to its implementation.^(6)^

Dobbs et al. conducted a systematic review to identify articles describing robotic assistance in plastic and reconstructive surgery.^(7)^ The review encompassed 68 articles assessing the advantages and disadvantages of robotics in these surgical fields. Specifically, 13 studies on the use of robotics in microsurgery were identified.^(7)^ These studies concluded that significant benefits, such as tremor elimination at the microsurgical level, were evident.^(4)^ However, the absence of specialized microsurgical instruments was a notable limitation. In muscle flap harvesting, traditionally performed with large incisions, robotics substantially reduced the incision size, rendering the procedure minimally invasive and leaving minor visible scars. Conversely, the video-assisted approach, akin to laparoscopy, is not widely accepted due to challenges in visualizing the operative field and the limitations of laparoscopic instruments. Transoral robotic surgery has emerged as the predominant domain for robotic-assisted procedures in plastic surgery, with at least 26 clinical studies documenting its application. Local reconstructive techniques include using the Facial Artery muscle-mucosal flap, frequently employed in reconstructing the mouth floor and soft palate. Additionally, other research indicates that robotic-assisted musculomucosal advancement flap pharyngoplasty produces favorable outcomes, reducing the risk of orocutaneous fistula and functional results.^(7)^

## OBJECTIVE

A scoping review was conducted to explore the emerging benefits of Robotic Surgery within the field of Plastic Surgery and its application in surgical practice.

## METHODS

**Figure 1:**
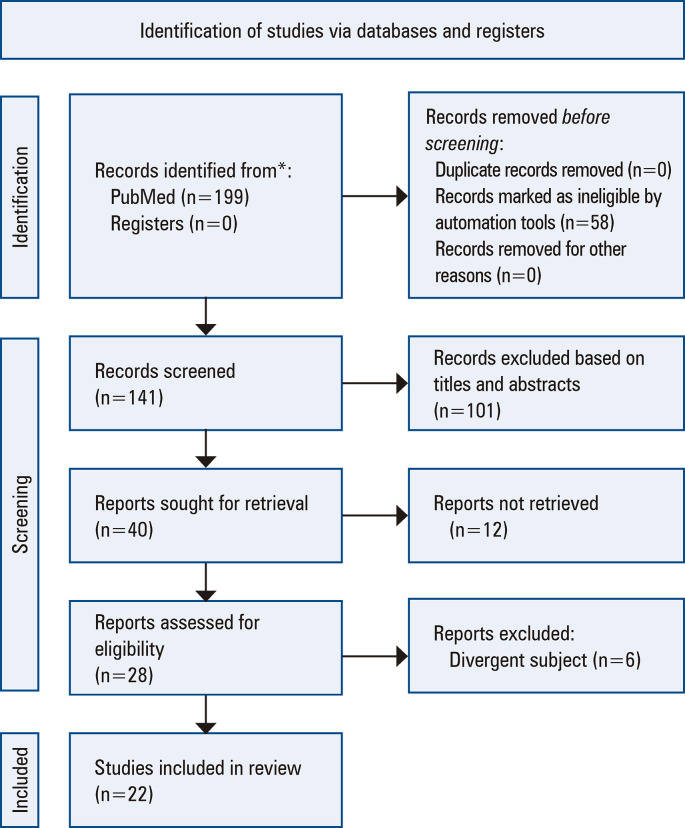
PRISMA flow diagram demonstrating the number of retrieved articles, those screened, and the final number included in the systematic review after completion of a full-text review * Consider reporting the number of records identified from each database or register searched (rather than the total number across all databases/registers).

Initially, the search terms “Robotic Surgery/Cirurgia Robótica” AND “Plastic Surgery/Cirurgia Plástica’’ were used in the PubMed and Cochrane databases in English and Portuguese. A total of 199 articles were found. A first screening was performed that excluded duplicates and irrelevant articles based on their titles and abstracts. A total of 40 articles remained. Further analysis of the articles and their methodologies was performed, leading to the exclusion of articles that did not focus on Plastic Surgery or were merely editorial articles without a defined scientific methodology (Figure 1). Ultimately, 22 articles were included in this study (Table 1). This study is cross-sectional, descriptive, qualitative, and non-experimental, with the capacity to formulate inferences for hypotheses.

**Table 1:** Studies related to the use of robotic-assisted plastic surgery that are eligible for the review

Authors	Year	Study design
General		
Gettman et al.^(3)^	2016	Literature review
Dy et al.^(4)^	2018	Literature review
Özkan et al.^(5)^	2019	Case report
Jimenez et al.^(6)^	2022	Systematic review
Dobbs et al.^(7)^	2017	Systematic review
Microsurgery		
Aitzetmüller et al.^(8)^	2022	Literature review
Ibrahim et al.^(9)^	2017	Literature review
Saleh et al.^(10)^	2015	Literature review
Breast reconstruction		
Kopkash et al.^(11)^	2020	Literature review
Yang et al.^(12)^	2021	Literature review
Vourtsis et al.^(13)^	2022	Literature review
Escandón et al.^(14)^	2022	Systematic review
Khan et al.^(15)^	2022	Literature review
Bishop et al.^(16)^	2021	Literature review
Toesca et al.^(17)^	2017	Randomized trial
Transoral surgery		
Balasundaram et al.^(23)^	2012	Literature review
Barrette et al.^(24)^	2022	Systematic review
Silverman et al.^(25)^	2022	Literature review
Park et al.^(27)^	2020	Meta-analysis
Lin et al.^(28)^	2022	Prospective randomized controlled trial
Longfield et al.^(29)^	2012	Literature review
de Almeida et al.^(30)^	2014	Literature review

## RESULTS

### Microsurgery

In the plastic surgery field, robotic surgery has been predominantly employed for microsurgery. Specifically, the Da Vinci - Intuitive^®^, the most commonly used operating system, was first used over 15 years ago for venous microsurgical anastomosis of the Deep Inferior Epigastric Perforator (DIEP) flap. However, the capacity of robotics in microsurgery has broadened with the introduction of platforms offering greater image magnification (up to 20:1) and enhanced tremor control. It is important to note that this progression has primarily occurred in the last five years.^(8)^

The use of robotics has increased the efficacy of lymphatic and lymphovenous anastomosis, offering considerable advances in managing lymphedema.^(8,9)^

The cost-effectiveness of robotic microsurgery in plastic surgery is not well-supported by studies with statistically significant findings. One of the critical limitations of Robotic Surgery is the initial expense associated with training and implementing robotic technology. This cost can be mitigated over time and with the adoption of the technique by multiple groups or specialties within a hospital.^(8-10)^ However, a formal and statistical cost-effectiveness study of robotic microsurgery in plastic surgery is yet to be carried out.

### Breast reconstruction

Robotic surgery has gained popularity in breast reconstruction, enabling surgeons to perform procedures such as implant-based reconstruction, tissue flap reconstruction, nipple reconstruction, and breast reduction with enhanced precision and control.^(11-15)^ This technology reduces scarring, diminishes pain, and expedites patient recovery times.

Robotic surgery offers significant benefits in reconstructive breast surgery. It allows surgeons to operate through smaller incisions, resulting in reduced scarring and more aesthetically pleasing results, such as with nipple-sparing techniques.^(11)^

The Food and Drug Administration (FDA) has not yet approved robotic-assisted nipple-sparing mastectomy (RNSM). However, a multi-center Investigational Device Trial is underway to secure 510k approval from the FDA for using robotics in nipple-sparing mastectomies.^(16)^ Robotic Surgery may enhance exposure during the procedure, potentially improving the preservation of vasculature to the mastectomy flap. Toesca et al. first described RNSM in 2015, when they performed the surgery through a single axillary incision and immediately placed an implant for reconstruction.^(17)^ Toesca et al. further reported a case series of 29 procedures with an average completion time of approximately 3 hours. The series demonstrated a low % conversion rate to open surgery of 6.9%, no major complications, and a rapid learning curve, typically requiring only 5 cases to achieve the average time.^(18)^

Existing endoscopic techniques for *latissimus dorsi* breast reconstruction have proven to be technically challenging, leading most surgeons to abandon them.^(19,20)^Selber first described a robotic harvest of the *latissimus dorsi* muscle through a cadaveric feasibility study.^(21)^ This study was followed up by a case series involving seven patients who underwent robotic *latissimus dorsi* muscle harvest using the previously mentioned technique.^(21)^ Five patients received pedicled breast reconstruction, and two had free flaps for scalp reconstruction. The study reported no major complications, and the robotic harvest time decreased significantly from two hours to one hour.^(21)^

The Transverse Rectus Abdominis Myocutaneous (TRAM) and Deep Inferior Epigastric Perforator (DIEP) flaps are commonly used techniques for breast reconstruction. The fascial incision for such procedures can be greatly minimized by approaching the pedicle dissection posteriorly, that can be facilitated by a robotic system.^(16)^

A randomized clinical trial comparing robotic mastectomy with open surgery in women with breast cancer yielded insightful results.^(22)^ On average, the total time for a unilateral surgery was 78 minutes longer for the robotic procedure than for the open procedure. However, complications from surgery were reported in 50% of patients in the open group, compared to 30% in the robotic group. More precisely, skin necrosis, including ischemia of the nipple-areola complex, was observed in 12.5% of patients after open surgery. In contrast, no skin or nipple necrosis was reported among the 40 patients undergoing robotic surgery.^(22)^

This randomized clinical trial also provided insight into patient satisfaction. Mean satisfaction scores for breasts and psychosocial well-being significantly increased from baseline at one year postoperatively in the robotic arm, while no significant change was observed after open surgery. Sexual well-being scores significantly decreased following open mastectomy but remained stable after robotic mastectomy. The nipple-areola complex (NAC) sensitivity was largely preserved post-robotics, with patients reporting less change in NAC sexual pleasure and describing the touch of the NAC as pleasant. Finally, patients in the robotic group were more likely to choose the same surgery again and recommend it to other women.^(22)^

Occasions may arise where a patient may undergo multiple robotic surgeries. As demonstrated above, a patient could undergo a robotic nipple-sparing mastectomy, followed by robotic DIEP reconstruction, which includes robotic microsurgical anastomosis. Additionally, the patient may receive a robotic lymphovenous bypass to manage lymphedema that may arise following axillary dissection.

### Transoral robotic surgery

Transoral robotic surgery (TORS) has gained popularity as a technique for tumor resection in the field of Head and Neck Surgery. It enables surgeons to excise tumors without needing external incisions, potentially preventing additional treatments. Transoral robotic surgery affords exceptional access to tumors in the upper aerodigestive tract, facilitating safe removal without resorting to mandibulotomy or lip-splitting incisions.^(23-25)^

In 2005, McLeod et al. performed the first transoral procedure on animal models, concluding that the robot provided advantages, including enhanced mobility, improved three-dimensional visualization, and reduced morbidity incisions.^(26)^ Since then, numerous institutions have published short-term data from small series trials. Long-term oncologic outcomes of the transoral robotic approach are not yet available due to limited sample sizes and follow-up durations. Nevertheless, these preliminary assessments indicate that minimally invasive resection effectively removes tumors and preserves patients’ swallowing function.

Park et al.^(27)^ conducted a systematic review and meta-analysis comparing the safety and effectiveness of TORS with traditional open surgery for treating oropharyngeal cancer. Analyzing 14 studies involving 2,265 patients, they demonstrated that TORS was associated with improved disease-free survival, shorter time to decannulation, reduced length of hospital stay, and a lower risk of free flap reconstruction. However, the study concluded that it was not possible to definitively determine the clinical safety and effectiveness, including functional and oncologic outcomes.^(28)^

Lin et al.^(28)^ evaluated the precision and safety of robot-assisted surgery in patients with mandibular deformity requiring mandibular contouring surgery. Their prospective randomized controlled study demonstrated that the robotic method achieved greater positioning accuracy and osteotomy plane angle precision compared to traditional surgery, enhancing bone shaving and safety.^(17)^

The promising outcomes prompted the FDA to approve TORS for treating select benign, T1, and T2 malignant head and neck tumors.^(29)^

Furthermore, robotic-assisted reconstructions have emerged as a viable option for surgeons treating larger head and neck tumors, where healing by secondary intention is often insufficient.^(23-25)^ Longfield et al. conducted a comprehensive review of the literature on TORS reconstruction, focusing on patient selection, tumor size, and location. The study concluded that TORS reconstruction is a safe and feasible technique that could expand the currently approved FDA scope. While healing by secondary intention is often the standard of care for T1 and T2 tumors, T3 and T4 tumors may likewise benefit from reconstruction after TORS, depending on the site and complexity of the lesion. Nonetheless, in many cases, healing by secondary intention provides a shorter recovery period and excellent functional outcomes. There is no clear guideline for determining when reconstruction after TORS is necessary versus when healing by secondary intention is sufficient.^(29)^

Transoral robotic surgery is a promising minimally invasive method for accessing the oropharynx to treat oropharyngeal defects. However, safety and health outcomes must be evaluated before implementation. To address this concern, de Almeida et al. developed a reconstruction algorithm for managing oropharyngeal defects based on the type of defect, the number of subsites involved, and complications such as exposure of the carotid artery, presence of pharyngocervical communication, and extensive palatal defect.^(30)^ As a result, they introduced the Classification for Oropharyngeal Robotic Defects (CORD) to categorize oropharyngeal defects and determine the most appropriate treatment pathway. Class I defects, involving only one subsite without complicating features, should heal by secondary intention for tonsils, pharynx, and tongue base defects or by primary closure for palatal defects. Class II defects, involving more than one subsite without complicating features, should heal by secondary intention for defects from the tongue base to tonsils or by musculo-mucosal flap closure for defects from tonsils to palates. Finally, class III and IV defects, involving only one subsite with complicating features and more than one subsite with complicating features, respectively, should undergo regional or free flap reconstructions.^(31)^

## DISCUSSION

Here, we examined and discussed the application of robotic surgery in Plastic Surgery, emphasizing its benefits, constraints, and prospective uses.

There are several vital points to consider regarding using robotic surgery in plastic surgery. First, it is crucial to outline the field-specific advantages of robotic surgery. These advantages include enhanced precision and accuracy by robotic surgical systems, allowing surgeons to perform intricate procedures with increased finesse. High-definition 3D imaging offers improved visualization, granting surgeons a detailed view of the surgical site. Robotic-assisted procedures typically result in less invasiveness, with smaller incisions leading to minimized scarring, reduced blood loss, and potentially shorter patient recovery times. Additionally, the ergonomic benefits, such as improved surgeon posture and reduced fatigue, warrant emphasis. Another advantage to discuss is the telemanipulation capabilities of robotic surgery, which can facilitate remote procedures and broaden patient access to specialized care.

Likewise, it is crucial to analyze the clinical outcomes and patient satisfaction of robotic surgery in plastic surgery procedures from the comparative studies evaluated here. We must assess factors including complication rates, revision rates, aesthetic outcomes, and patient-reported satisfaction. Additionally, functional outcomes, such as enhanced muscle function and range of motion, are particularly important in reconstructive procedures. The long-term results and the durability of outcomes over time warrant examination. This review highlights the scarcity of articles and research involving robotics in plastic surgery, indicating that its use is still in the early stages.

In contrast, robotic surgery has been employed in microsurgery for microsurgical venous anastomoses and shows potential in facilitating lymphatic/lymphovenous anastomoses, benefiting lymphedema treatment. However, studies on the cost-effectiveness of this approach remain scarce.

Robotic surgery is becoming an increasingly popular option for breast reconstruction as it allows for complex procedures to be performed with greater precision and control, resulting in reduced scarring, less pain, and faster patient recovery. This technique can also be applied in nipple-sparing mastectomies, reducing the need for external incisions and preserving the aesthetics of the nipple.

Transoral robotic surgery is widely used for tumor resection in head and neck surgeries. The enhanced flexibility and access provided by the single-port robotic system improves ergonomics for the surgeon, facilitates muscle dissections, and offers safer access to tumors in the upper aerodigestive tract without necessitating additional external incisions. Furthermore, TORS has replaced several surgical procedures traditionally performed via transoral microscopic or open cervical approaches. Studies have demonstrated that TORS results in lower estimated blood loss, reduced hospital stay lengths, and fewer postoperative complications compared to open approaches,^(32)^ particularly in the pediatric population, which benefits from the approach due to its unique oral cavity anatomy^(33)^ in procedures such as palatoplasty.

The most notable limitation of current robotic systems is their restricted spatial flexibility. Compared to laparoscopic applications, the head and neck region, characterized by narrower triangulation angles, requires a robot that can navigate confined spaces. By adapting the robotic configuration to employ a single access port for the camera and working arms, these deeper areas are now more accessible. Consequently, with enhanced transoral access to areas beyond the oropharynx, a broader spectrum of applications is anticipated.

Next, addressing the challenges and limitations associated with robotic surgery is crucial. These challenges include the costs of acquiring and maintaining robotic surgical systems and the need for specialized training to enable surgeons to become proficient. Additionally, the accessibility and availability of robotic surgical systems warrant discussion, as not all healthcare facilities can access this technology.

Future directions and emerging applications of robotic surgery within plastic surgery could include aesthetic procedures, such as facial rejuvenation, body contouring, and rhinoplasty. Additionally, integrating artificial intelligence with robotic surgery systems merits attention, as it offers the potential for automated tissue recognition, enhanced surgical planning, and support for intraoperative decision-making.

Ethical considerations are crucial in the discourse on robotic surgery in plastic surgery. Issues such as patient autonomy, informed consent, and the effects on the doctor-patient relationship must be addressed. Additionally, the ethical responsibilities of surgeons to adopt and promote robotic surgery, focusing on patient safety and welfare, should be underscored.

Robotic surgery’s future in plastic surgery likely includes broadening its use in aesthetic procedures and incorporating artificial intelligence to automate tissue recognition and surgical planning. Addressing ethical issues related to patient autonomy, informed consent, and the doctor-patient relationship is also crucial. Although scientific literature on robotic surgery in plastic surgery is currently sparse, its potential for expansion and progress is evident. The case of a patient receiving a robotic nipple-sparing mastectomy, with subsequent robotic reconstruction and lymphovenous bypass, exemplifies the prospective advancements in robotic surgery within the field.

## CONCLUSION

Robotic surgery in plastic surgery, though young, shows considerable potential for the future. It offers enhanced precision, improved visualization, reduced invasiveness, and ergonomic advantages for surgeons. Robotic surgery also opens the possibility for telemanipulation, allowing for remote procedures and broadening access to specialized care. Comparative studies indicate favorable outcomes regarding complication rates, aesthetic results, and patient satisfaction. Nevertheless, the integration of robotic surgery is hindered by challenges, including high costs, the need for specialized training, and limited availability in healthcare facilities.
